# An algorithm to identify rheumatoid arthritis in primary care: a Clinical Practice Research Datalink study

**DOI:** 10.1136/bmjopen-2015-009309

**Published:** 2015-12-23

**Authors:** Sara Muller, Samantha L Hider, Karim Raza, Rebecca J Stack, Richard A Hayward, Christian D Mallen

**Affiliations:** 1 Arthritis Research UK Primary Care Centre, Institute for Primary Care & Health Sciences, Keele University, Keele, UK; 2 Centre for Translational Inflammation Research, School of Immunity and Infection, University of Birmingham, Birmingham, UK; 3 Department of Rheumatology, Sandwell and West Birmingham Hospitals NHS Trust, Birmingham, UK

**Keywords:** PRIMARY CARE, EPIDEMIOLOGY, RHEUMATOLOGY

## Abstract

**Objective:**

Rheumatoid arthritis (RA) is a multisystem, inflammatory disorder associated with increased levels of morbidity and mortality. While much research into the condition is conducted in the secondary care setting, routinely collected primary care databases provide an important source of research data. This study aimed to update an algorithm to define RA that was previously developed and validated in the General Practice Research Database (GPRD).

**Methods:**

The original algorithm consisted of two criteria. Individuals meeting at least one were considered to have RA. Criterion 1: ≥1 RA Read code and a disease modifying antirheumatic drug (DMARD) without an alternative indication. Criterion 2: ≥2 RA Read codes, with at least one ‘strong’ code and no alternative diagnoses. Lists of codes for consultations and prescriptions were obtained from the authors of the original algorithm where these were available, or compiled based on the original description and clinical knowledge. 4161 people with a first Read code for RA between 1 January 2010 and 31 December 2012 were selected from the Clinical Practice Research Datalink (CPRD, successor to the GPRD), and the criteria applied.

**Results:**

Code lists were updated for the introduction of new Read codes and biological DMARDs. 3577/4161 (86%) of people met the updated algorithm for RA, compared to 61% in the original development study. 62.8% of people fulfilled both Criterion 1 and Criterion 2.

**Conclusions:**

Those wishing to define RA in the CPRD, should consider using this updated algorithm, rather than a single RA code, if they wish to identify only those who are most likely to have RA.

**Strengths and limitations of this study**

- An original, but out-of-date, definition of rheumatoid arthritis derived from validated data is updated.
- A large sample of high-quality, representative primary care data was available to test the updated algorithm.
- A comparison is made between the original definition and the updated algorithm.
- The updated algorithm could not be compared to full medical records.

## Introduction

Rheumatoid arthritis (RA) is a common inflammatory multisystem disorder involving joint inflammation, and increased morbidity and mortality from related conditions, for example, cardiovascular disease.^1^ Delays in identifying and treating RA are common and are associated with worse outcomes. Research into RA has been focused in secondary care (eg, early arthritis clinics to identify patients in the earliest stage of disease). Algorithms and criteria to define RA developed in secondary care settings have also been developed. However, there are likely to be aspects of the disease that it is not possible to fully investigate in secondary care alone. For example, primary care studies are likely to be needed to determine healthcare usage prior to diagnosis,^2^ or whether patients receive screening for diseases for which they are at high risk.^3^

One potential way of investigating RA in primary care is the use of healthcare databases, for example the Clinical Practice Research Datalink (CPRD), QResearch or The Health Improvement Network (THIN). Use of such databases in epidemiological research is increasing, with CPRD data used in over 190 studies in 2014.^4^ These data sources include data recorded in routine clinical practice, such as information regarding symptoms, diagnoses, prescriptions and referrals. These large databases are highly generalisable, because they cover large numbers of people from the general population (eg, CPRD covers approximately 6% of the UK population^5^), meaning that they can be used efficiently in epidemiological studies.

Use of these databases requires accurate identification of the conditions or treatments of interest. In the UK, this is often carried out using a clinical coding system such as Read codes, or for medications, British National Formulary codes. However, the use of single codes is not always suitably sensitive and/or specific and sometimes more complex algorithms to define a disease or treatment of interest are needed.

A definition has previously been developed to accurately identify highly probable cases of RA in primary care medical records,^6^ specifically in the General Practice Research Database (GPRD, now the CPRD). This definition used a combination of diagnostic Read codes and prescription records to define a patient as having or not having RA, achieving sensitivity of 84% and specificity of 86%. However, this work was conducted in data from 1987 to 2002 and since then the Read code dictionary has been updated and extended, and importantly, a new class of treatments for RA, known as biologics, has been introduced. This means that the original definition is now somewhat dated. Therefore, the aim of this study was to describe our updating of the definition of Thomas *et al*^6^ in order to create an up-to-date algorithm to identify highly probable RA cases in the CPRD and to compare the characteristics of the algorithm to the original.

## Methods

### The original Thomas algorithm to define RA

The original algorithm for RA was developed in the GPRD, a predecessor to the current CPRD. In order to derive this algorithm, 224 patients with at least one diagnostic Read code for RA were randomly selected from the GPRD and their full, anonymised medical records reviewed to ascertain whether they did indeed have RA.^6^ Coded entries of symptoms, diagnoses and prescriptions were then assessed and compared to the classification of RA from the full notes review, using a multivariable logistic regression model. This resulted in the algorithm to define a case of RA (box 1).

Box 1 **Thomas *et al*^6^ algorithm for rheumatoid arthritis (RA) in the General Practice Research Database**

Criterion 1: At least one diagnostic Read code for RA and at least one appropriate prescription of a disease modifying antirheumatic drug (DMARD) with no alternative indication for the DMARD;

or

Criterion 2: all three of the following:

- a) two or more diagnostic Read codes for RA (on different dates);
- b) no alternative diagnosis after the final RA code;
- c) RA code in group 1 (seropositive or erosive RA) or group 2 (‘rheumatoid arthritis’ codes eg, RA of knee), opposed to only group 3 (systemic manifestations of RA) or group 4 (seronegative RA or other weak evidence of RA).

### Updating the Thomas algorithm

#### RA-related codes

Starting with the list of RA Read codes classified as groups 1, 2, 3 and 4 by Thomas *et al*,^6^ the CPRD Medical Dictionary was used to look up key terms associated with each code (eg, ‘rheumatoid’, ‘felty’, ‘still's’) until all codes on the original list would have been found if the code list remained the same. The new list of codes was then reviewed, and using the original severity grouping as a guide, the new list of codes was grouped by severity. This process was conducted by a consultant rheumatologist (SLH) and a non-clinical researcher (SM).

#### Drugs used to treat RA

The list of drugs considered to be used to treat RA in the original algorithm was not available from the authors. Therefore, the British National Formulary (BNF) was reviewed to identify all drug specified as being for the treatment of ‘rheumatoid arthritis and other inflammatory disorders’ within the musculoskeletal system and joint diseases chapter. This list was then reviewed by SLH to ascertain whether this list covered all drugs used in clinical practice and that all of the drugs identified were relevant to RA. Oral steroids and non-steroidal anti-inflammatory drugs were excluded, as they were treated separately when the original algorithm was developed, and were found to be insufficiently specific to a diagnosis of RA.^6^

Alongside this list of potential RA treatments, which consisted of conventional and biological disease modifying antirheumatic drugs (DMARDs), a list of potential alternative indications each for these treatments was compiled from the BNF. Synonyms for these conditions were then established and the CPRD Medical Browser used to assemble a list of potentially relevant codes, which was reviewed by SLH and CDM (professor of general practice), and consensus reached.

#### Alternative diagnoses

As with drugs used to treat RA, a list of codes that would indicate a diagnosis that supersedes RA was not available from the authors of the original algorithm. Therefore, a list of potential conditions and their synonyms was reached by consensus between SLH and CDM. The CPRD Medical Browser was searched for these terms to establish a list of codes and related terms, which was then reviewed by SLH and CDM in order to determine a final list of codes indicating an alternative diagnosis to RA.

### Study sample

For this study, a sample of all individuals with a first RA-related Read code (codes in groups 1–4, as defined above) between 1 January 2010 and 31 December 2012, was obtained from the CPRD. RA status was determined according to the definition described above (box 1). The full period of the record held by the CPRD was downloaded for all individuals in the sample, before and after their first RA code.

### Statistical analyses

Absolute numbers and percentages were used to show the proportion of people with an RA code who were subsequently defined as having ‘definite’ RA according to the updated algorithm. Analyses were repeated separately in gender-specific and age-specific groups (grouped roughly into quartiles according to the distribution in the data: <50 years; 50–59 years; 60–69 years; ≥70 years) and year of first RA code.

Analyses were repeated for individual criteria within the algorithm for RA.

## Results

### Updated lists of Read codes to apply in Thomas algorithm

#### RA-related codes

The search of key terms from the original list of Read codes produced a larger number of codes. Some codes were not relevant and were excluded (eg, family history of RA). Of the remaining codes, some had the same attached terms as codes in the original list, while others were new, and clinical judgement (SLH/CM) was used to assign them to a severity group.

#### DMARDs used to treat RA

A full list of the DMARDs licensed for the treatment of RA in the UK at the time of the study (January 2014), was compiled from the BNF. The other licensed uses or alternative indications for these drugs were assessed using the BNF. ‘Alternative indications’ for these DMARDs varied by substance, but included psoriatic arthritis, seronegative spondyloarthropathy, juvenile idiopathic arthritis, psoriasis, inflammatory bowel disease, systemic lupus erythematosus, transplant, vasculitis, leukaemia and lymphoma. Code lists to define each of these conditions were formulated, with consensus on the final list reached between SLH and CDM.

#### Alternative diagnoses

Alternative diagnoses to RA (ie, those which if present after the final RA code in the record would supersede a diagnosis of RA), were decided to be psoriatic arthritis, ankylosing spondylitis and other spondyloarthropathies. Polymyalgia rheumatica (PMR) was also considered as a potential alternative diagnosis, as RA would be an alternative diagnosis for PMR. However, it was decided that as PMR is often considered a diagnosis of exclusion, this was not appropriate.

Full lists of the codes used to define RA, DMARDs and their alternative indications and alternative diagnoses are available from the clinicalcodes.org website and in the authors’ institutional repository (keele.ac.uk/mrr). Searches for appropriate codes to implement the algorithm were conducted in all available data for each individual.

### Proportion of those with RA code considered to have definite RA

Between 2010 and 2012, 4161 people were identified in the CPRD as having a first Read code for RA. The median length of time from the index date (date of first RA code) to the final consultation in the record of these patients was 3.25 years (IQR 2.5, 4.1), and the median length of the consultation record prior to the index date was 37.7 years (25.4, 49.0). Of these, 3577 (86%) were considered to have definite RA according to the updated algorithm (table 1). A total of 659 (15.8%) people met only the first criterion of a DMARD with no alternative indication. A total of 304 (7.3%) people satisfied the second set of criteria only (ie, ≥2 RA codes on separate dates, no alternative diagnosis after final RA code and an RA code in severity group 1 or 2). A total of 2614 (62.8%) people met both sets of criteria.

**Table 1:** Fulfilment of each RA definition by the sample, compared to Thomas *et al*^6^ in GPRD

	Thomas *et al*^6^ N=31 830	Current sample N=4161
Database	GPRD	CPRD
Time frame	1987–2002	2007–2012
Age of sample	≥16 years	≥18 years
Criterion 1: appropriate DMARD prescription	15 746 (49)	3273 (78.7)
Criterion 2: all 3 of the following	–	2918 (70.1)
>1 RA code during follow-up	16 300 (51)	3230 (81.5)
No alternative diagnostic code after last RA code	27 184 (85)	4109 (98.8)
≥1 RA code in group 1 or 2	27 738 (87)	3535 (89.2)
Full diagnostic algorithm (criterion 1 and/or criterion 2)	19 492 (61)	3577 (86.0)

–, Data not available.

CPRD, Clinical Practice Research Datalink; DMARD, disease modifying antirheumatic drug; GPRD, General Practice Research Database; RA, rheumatoid arthritis.

Males and females with an RA code were equally likely to meet the definition of RA (p=0.369; table 2). There was however a difference in the rate of ‘definite diagnosis’ across age groups, with those aged 60–69 years most likely to meet the definition (88%), and those aged <50 years least likely (83.8%) (p=0.010). Similar patterns were seen across age groups within each gender as were seen overall, although males were most likely to have definite RA in the 50–59 years age group. On the whole, the proportion of people with a single RA code meeting the updated definition of RA was relatively stable across the 3 years included in this study, although the definition of RA was less likely to be met in those receiving their first RA code in 2011 (88%), with slightly lower rates of confirmed diagnosis in earlier and later years (p=0.029). This difference is driven by a combination of differences in the number of people with a suitable DMARD and the number of people with multiple RA codes (table 3).

**Table 2:** Fulfilment of the RA definition by age and gender

n (%)	All	Males	Females
All	3577 (86.0)	1188 (85.3)	2389 (86.3)
<50 years	902 (83.8)	231 (81.1)	671 (84.8)
50–59 years	786 (87.6)	225 (88.9)	561 (87.1)
60–69 years	942 (88.0)	356 (87.9)	586 (88.1)
≥70 years	947 (84.7)	376 (83.6)	571 (85.5)

RA, rheumatoid arthritis.

**Table 3:** Fulfilment of the RA definition by year of first RA code

n (%)	Full diagnostic algorithm	Criterion 1	Criterion 2	>1 RA code during follow-up	No alternative diagnostic code after last RA code	≥1 RA code in group 1 or 2
2010	1204 (84.6)	1101 (77.4)	967 (68.0)	1050 (79.9)	1404 (98.7)	1186 (90.3)
2011	1186 (88.0)	1096 (81.3)	969 (71.9)	1068 (82.0)	1327 (98.4)	1173 (90.0)
2012	1187 (85.4)	1076 (77.4)	982 (70.7)	1112 (82.7)	1378 (99.1)	1176 (87.4)
p Value	0.029	0.016	0.068	0.163	0.246	0.033

Full algorithm requires meeting either criterion 1 or criterion 2 (or both); criterion 2 requires having (a) >1 RA code during follow-up and (b) no alternative diagnostic code after last RA code, and (c) ≥1 RA code in group 1 or 2.

RA, rheumatoid arthritis.

## Discussion

Accurate diagnosis of RA is of paramount importance clinically, as current guidelines recommend early and aggressive treatment with DMARDs. In order to take this approach clinically, further research will be necessary to accurately identify patients with RA in primary care. This updated algorithm could contribute to this research. Without suitable means of defining an RA cohort that has a high probability of being true RA, such studies would be of poorer quality. This study has updated the definition, initially proposed by Thomas *et al*,^6^ to define RA in the GPRD for use in the CPRD. The original authors of this definition stated that the use of their algorithm prior to 2002 appeared to be valid, but that it would need to be updated for future work, specifically around the use of biological therapies. The current study has made this update, without unnecessarily complicating the algorithm by attempting to recreate it from first principles. Using this updated algorithm, 86% of people with a code for RA were considered to have ‘definite’ RA.

Thomas *et al*^6^ studied 258 people aged 16 years and over with a code for RA in the GPRD. After correspondence with the patients’ general practitioners (GPs) and review by expert rheumatologists, they considered 125 (48%) of these people to have definite RA. We could not make this comparison in the current study, as we did not have access to full medical records for people with an RA Read code. Hence, we are not able to report formal assessments of the algorithm's performance, such as sensitivity or specificity. Instead, the current study sought to update the algorithm previously developed by Thomas *et al*^6^ and compare it to the original. In the original study, the authors found that of the 31 830 people that they identified as having an RA code in their GPRD record, 61% met the definition of RA. This compares to 86% in the current study, suggesting that the updated algorithm may be more sensitive, or less specific than the original. However, we believe that this higher rate of confirmed RA diagnoses may reflect changes in coding practice over time, or that GPs are less willing to code RA in the medical records until the diagnosis is confirmed by a specialist. This may mean that a single code for RA is now a more accurate reflection of a true diagnosis of RA than was previously the case.

Consideration of the specific elements of the definition, in comparison to the work of Thomas *et al* showed that the largest difference came from the proportion of people with a record of a DMARD with no alternative indication. This criterion, which in the presence of a single RA Read code, was sufficient to classify someone as having definite RA, was met by 78.7% of the current sample, opposed to 49% of Thomas *et al*'s^6^ sample. This may reflect the updated list of DMARD codes in the current study, but given the different time frames of the data sets, is likely due to the move to transfer repeat prescribing of DMARDs from secondary to primary care in the UK. The current study also saw a substantial increase in the number of people with more than one RA code in the study period (51% vs 81.5%), and indeed this increase may be larger than it first appears, when the length of follow-up time in the studies is considered; up to 5 years in the current study compared to up to 16 years in the original. It seems likely that the increase in DMARD recording and the number of RA codes, combined with an increase in the number of people without an alternative diagnosis after their final RA code, reflects general changes in coding practice, with codes becoming more specific and less likely to be entered into the record until GPs are confident of the diagnosis. It could also reflect a change in the diagnostic process used by rheumatologists since the introduction of the 2010 American College of Rheumatology/European League Against Rheumatism classification criteria for RA,^7^ which mean that rheumatologists are likely to diagnose RA earlier in the disease course, and therefore GPs may in turn code it earlier. Similarly, the introduction of the National Institute for Health and Care Excellence Rheumatoid Arthritis Guideline in 2009 should have prompted faster referral by GPs of suspected patients with RA to secondary care. Thereby speeding up, and potentially increasing the accuracy of the diagnoses recorded in primary care records such as the CPRD.

However, we cannot rule out the possibility that changes over time in coding practice and in the management of RA could mean that the necessary components of a definition of RA may have changed, and we did not consider this in the current study. For example, in the original study, Thomas *et al*^6^ considered joint symptoms/investigation codes after the first RA code, and the presence of two or more non-steroidal anti-inflammatory prescriptions in a 6-month period as potential predictors of true RA, but they were not considered optimal for the final model. To recreate the whole process from the original formulation of this definition of RA would be hugely intensive in terms of financial and human resource, and seems unlikely to yield a vastly different model. The current study therefore presents a necessary and efficient update to the existing work in this area that can be readily applied in research practice.

For the reasons discussed above, those wishing to apply the updated algorithm should do so with caution, particularly in the situation where a highly sensitive definition of RA is required (eg, prevalence study, clinical audit). The current algorithm is likely to be unsuitable for such studies, as it is designed to find those with highly probable RA. Indeed, if changes in coding practice have occurred in the manner discussed above, with GPs more certain of a diagnosis before entering a code, the updated algorithm may be more specific than the original. Before the algorithm is used in settings where a less specific definition of RA is required, it would be sensible to formally test its performance, by comparing to full medical records, as was the case in its original development. However, this was beyond the scope of the current study.

In addition to the potential weaknesses of this study discussed above, there are some limitations to the use of clinical databases in general that should be considered in all such studies. These include a reliance on what is coded by the general practice, which may be different to the patient's perception of the consultation, and indeed may not reflect the entire content of a consultation. This is particularly the case when considering symptoms, opposed to clear-cut diagnoses, but is less of a problem with prescriptions, which are generally issued electronically and therefore recorded by default. In addition, it is usually not possible to understand the reasons for a particular diagnostic code or prescription being recorded and one must rely on what is in the record having been a true event and accept that anything that is not present did not happen.

Our investigation of the proportion of people fulfilling the definition RA according to the year was intended to investigate the algorithm's stability over time. However, it also gave some insight into the time required to fulfil the criteria (eg, second RA code). The stability of the proportion fulfilling the definition over time suggests that 12 months seems a reasonable time frame in which to consider follow-up after the first RA code, in order to apply this definition.

If GPs are waiting to code a diagnosis of RA until they are confident that this is the correct diagnosis, for example, when it is confirmed by a specialist, this has implications for studies requiring a ‘start time’ when a condition was suspected by the GP, for example, those wishing to look at care pathways, or early symptoms, the time of the first diagnostic code will be much later than the period of real interest. This is issue that has already been raised by others,^2^ ^8^ and indeed was investigated in relation to RA by Nicholson *et al*^2^ who suggested a range of ‘indicator markers’ for early inflammatory arthritis. This is something that researchers may wish to consider in applying this new updated algorithm for RA in practice, dependent on their research question.

This updated algorithm for RA in the CPRD could be applied in other studies in the CPRD and indeed in other databases. Researchers should be aware of the follow-up time available after an RA code in which an individual can fulfil the definition of RA. Further research in this field, should resources allow, might consider testing this updated algorithm for RA against full medical records.

A strength of the current study was that it was careful to exclude the period when RA was included in the Quality and Outcomes Framework (QOF) a set of quality standards by which UK GPs receive some of their funding. In 2013–2014, RA was included in the QOF, requiring GP to maintain a register of patients, provide them with a face-to-face review and dependent on their age, screen them for cardiovascular disease and fracture risk. This package of care was worth 18 QOF points. In the following and subsequent years, this was reduced to only the register and review and worth only six points. The inclusion of a condition in QOF has this has been known to alter the way in which GPs code the conditions and indeed we found that the number of individuals with a new RA code was considerably higher in 2013–2014 than in the years before or after. Future studies should exercise caution if including this 1 year period in their work, as the algorithm has not been tested in this setting.

Although diagnoses recorded in the CPRD have been shown in general to be valid,^9^ further work to develop definitions of specific conditions should be compiled and made openly available. This would increase the credibility of work in the field and enable more effective use of these rich resources, especially where diagnosis and/or management is largely primary care based.

This study has updated a definition of RA in a large representative database of primary care medical records from the UK, which can be applied in a range of studies, where this condition is a key outcome or exposure, or indeed where it is of interest as a confounding or effect modifying factor. Future studies of RA in primary care databases should use this updated definition, rather than the original version.
